# Predicting Risk for Patent Ductus Arteriosus in the Neonate: A Machine Learning Analysis

**DOI:** 10.3390/medicina61040603

**Published:** 2025-03-26

**Authors:** Ana Maria Cristina Jura, Daniela Eugenia Popescu, Cosmin Cîtu, Marius Biriș, Corina Pienar, Corina Paul, Oana Maria Petrescu, Andreea Teodora Constantin, Alexandru Dinulescu, Ioana Roșca

**Affiliations:** 1 Department of Obstetrics and Gynecology, “Victor Babeş” University of Medicine and Pharmacy, Eftimie Murgu Sq. No. 2, 300041 Timişoara, Romania; cristina.jura@umft.ro (A.M.C.J.);; 2 Medici’s MedLife Hospital Timișoara, Ciprian Porumbescu Street No. 9, 300236 Timișoara, Romania; 3 2nd Pediatrics Clinic, Department of Pediatrics, “Victor Babes” University of Medicine and Pharmacy, 300041 Timisoara, Romania; 4 Pediatric Cardiology, Clinical Hospital of Obstetrics and Gynecology “Prof. Dr. P.Sirbu”, 060251 Bucharest, Romania; 5 Doctoral School, University of Medicine and Pharmacy “Carol Davila”, 020021 Bucharest, Romania; 6 Neonatology Department, Clinical Hospital of Obstetrics and Gynecology “Prof. Dr. P.Sirbu”, 060251 Bucharest, Romania

**Keywords:** patent ductus arteriosus, neonatal risk, machine learning, maternal pathology, neonatal outcomes

## Abstract

*Background and Objectives*: Patent ductus arteriosus (PDA) is common in newborns, being associated with high morbidity and mortality. While maternal and neonatal conditions are known contributors, few studies use advanced machine learning (ML) as predictive factors. This study assessed how maternal pathologies, medications, and neonatal factors affect the risk of PDA using traditional statistics and ML algorithms: Random Forest (RF) and XGBoost (XGB). *Materials and Methods*: A retrospective 3-year cohort study of 201 NICU neonates assessed maternal and neonatal factors. Logistic regression (LR) and chi-square analyses identified significant predictors, while ML models enhanced predictive accuracy and pinpointed key PDA factors. *Results*: LR identified prolonged rupture of membranes (>18 h) as the most significant predictor (OR: 13.03, *p* < 0.001). The ML models identified gestational age, maternal anemia, prenatal care level, birth weight, prolonged rupture of membranes, medication usage, diabetes, pregnancy-induced hypertension, SARS-CoV-2 infection, and cervical cerclage as key predictors. The RF model had 76.3% accuracy, moderate sensitivity (47.4%), and high specificity (90%). XGB performed better with 81.4% accuracy, an AUC of 0.872, sensitivity of 92.5%, and specificity of 57.9%. *Conclusions*: This study shows that maternal and neonatal factors significantly influence the risk of PDA. ML, particularly XGBoost, enhances predictive abilities, guiding targeted interventions and improving neonatal outcomes.

## 1. Introduction

Persistent ductus arteriosus (PDA) is a common disorder in newborns and a significant predictor of morbidity and mortality. Several studies have been conducted to identify potential factors associated with delayed ductal closure in term newborns [1], but reports of maternal factors associated with early PDA in newborns are limited [2,3].

The ductus arteriosus (DA) is a fetal structure that connects the main pulmonary artery and the aorta. Its main function is to shunt blood flow away from the pulmonary circulation, given that the vascular resistance in the pulmonary circulation is high, and toward the aorta, closer to the peripheral organs with lower vascular resistance. To provide flow-dependent vasodilation capability and the large variability in postnatal shunt size necessary for adapting to the systemic vascular resistance, the DA’s structure is characterized by a uniformly developed middle layer of longitudinal smooth muscle cells, which acquire the capability of constriction after birth [4]. In the human fetus, the previously described DA function results in left-to-right shunting of oxygenated blood from the aortic arch to the main pulmonary artery. After birth, the basic switch from a shunt to a reentry system and the consequent closure of the DA lead to right-to-left shunting of deoxygenated blood from the main pulmonary artery to the aorta, thereby increasing the amount of oxygenated blood sent toward the body [5].

Traditionally, there is a tendency to treat neonatal PDA, and attention is urgently required. However, those treated pharmacologically alone are limited, and there are more complicated outcomes [6]. Compared to mothers with pathological pregnancy, the risk of PDA is also higher for these newborns. However, there are few studies of PDA association with pathologic pregnancy, such as non-infectious inflammatory diseases and autoimmune-related diseases [7,8]. Although the outcome is neonatal hypoxia, the mother’s illness has not been relevant. Focusing on the fact that the fetus responds to maternal symptoms of hypoxia, the fetus of pathologic pregnancy, including maternal trauma, may exacerbate fetal damage by maternal hypoxia [9].

Maternal hypertension impacts fetal circulatory development, potentially resulting in hypertrophy of left-heart structures and affecting the risk of patent ductus arteriosus due to modified blood flow and vascular resistance [10,11,12]. Also, it is known that gestational diabetes and type 1 diabetes in pregnancy are linked to hyperglycemia, which may affect the cardiac development of neonates, raising the risk of PDA in offspring [13,14]. Maternal thyroid dysfunction, especially hypothyroidism, may impair fetal vascular physiology [15], potentially postponing ductus arteriosus closure and elevating the risk of patent ductus arteriosus due to heightened prostaglandin levels and alterations in vascular resistance [16,17]. Other potential maternal contributing factors to PDA include infections, particularly urinary tract infections during pregnancy [18,19], but also viral infections during pregnancy, and maternal anemia, specifically iron deficiency anemia [20,21,22,23].

Machine learning enables machines to learn from a training dataset and predict outcomes for new data. It uses statistical theory to build mathematical models, aiming to derive inferences from samples [24]. Decision trees are classification techniques in data mining and machine learning, using a straightforward algorithm easily understood by researchers. As white-box models, they offer non-parametric flexibility, handle heterogeneous data, and classify sequential data without needing normalized features. They also require less execution time for classification than other methods [25]. Machine learning models, such as Random Forest and XGBoost, significantly surpass traditional statistical methods. They adeptly manage intricate, nonlinear relationships among predictors, delivering improved predictive accuracy and valuable insights into clinical risk evaluation. The Random Forest (RF) predictive model, based on decision trees, is a behavioral analysis tool that manages extensive datasets from modern supply chain operations, especially in healthcare [26]. The RF model is highly precise among common data classification algorithms and can handle extensive datasets with specific parameters. It efficiently manages diverse variables, making it suitable for complex tasks like health supply chain management. If a class is less prevalent, datasets can be automatically balanced [27]. The Extreme Gradient Boosting (XGBoost) model is prominent in machine learning for its generalizability, low overfitting risk, and high interpretability. It excels in predictive medicine tasks using tabular data like electronic health records and is used in critical care scenarios [28].

Machine learning has shown remarkable potential in cardiology. Studies have effectively used ML models to predict obstructive coronary artery disease using electrocardiographic features from treadmill exercise tests and applied deep learning techniques to accurately predict short-term mortality in acute pulmonary embolism. These precedents highlight ML’s capability in predictive cardiology, informing our decision to apply these methodologies in predicting neonatal PDA risk [29,30].

The study seeks to identify critical predictive features and evaluate the relative efficacy of algorithms by analyzing an extensive dataset of maternal and neonatal health indicators, focusing on accuracy, sensitivity, specificity, and additional diagnostic metrics. The primary objective is to develop a dependable predictive instrument to aid healthcare professionals in the early detection of neonates at elevated risk for PDA, thereby enabling prompt interventions and enhancing neonatal care outcomes.

## 2. Materials and Methods

A retrospective analysis was conducted based on a cohort of both preterm and term newborns who were hospitalized in the Neonatal Intensive Care Unit (NICU) in the Clinical Hospital of Obstetrics and Gynecology “Prof. Dr. Panait Sârbu” of Bucharest, Romania. We included all newborns admitted to the NICU between 1 January 2021 to 31 December 2023, over a period of three years. Ethical approval was obtained from the Research Ethics Committee of the Clinical Hospital of Obstetrics and Gynecology “Prof. Dr. Panait Sârbu” of Bucharest, no. 16516/11 December 2024. The dataset included a total of 201 cases with detailed maternal and neonatal variables.

The inclusion criteria covered all neonates admitted to the NICU during the study, provided there was comprehensive documentation on maternal health conditions and neonatal outcomes. Exclusion criteria applied to neonates missing essential clinical data, such as gestational age, birth weight, maternal pathology, medication usage, or diagnostic information. The diagnosis of patent ductus arteriosus (PDA) was confirmed through echocardiographic assessment, revealing left-to-right shunting, ductal diameter, and signs of hemodynamic implications.

### 2.1. Variables and Data Preprocessing

Data were preprocessed to convert categorical variables into a suitable format for analysis. Key predictor variables included maternal age, prenatal care level, specific maternal pathologies such as hypertension, diabetes, infections, and medications taken during pregnancy (antibiotics, antihypertensives, and steroids). Neonatal outcomes included PDA diagnosis as the primary target variable (binary variable indicating presence or absence of PDA). For predictor variables, we included maternal and medication variables, as follows:

- Maternal Pathologies: Conditions such as hypertension, diabetes, anemia, infections (e.g., GBS, SARS-CoV-2), and pregnancy complications (e.g., preeclampsia, prolonged rupture of membranes).
- Medication During Pregnancy: Administration of medications, including aspirin, nifedipine, antibiotics, and insulin.

Maternal health variables selected for analysis included hypertension, diabetes, anemia, Group B Streptococcus (GBS) infection, SARS-CoV-2 infection, preeclampsia, and prolonged rupture of membranes (PROM). These variables were chosen for their established clinical significance, as documented in medical literature, influencing fetal cardiovascular development, neonatal morbidity, and particularly, the risk of Patent Ductus Arteriosus. Specifically, maternal hypertension, diabetes, and preeclampsia impact fetal hemodynamics and cardiac function. Anemia and prolonged rupture of membranes are linked to altered fetal oxygenation and inflammatory responses, potentially affecting ductal closure mechanisms. Infections like GBS and SARS-CoV-2 have systemic inflammatory effects that can influence neonatal cardiovascular physiology and ductal patency. The selected medications—aspirin, cefuroxime, methyldopa, enoxaparin, amoxicillin with clavulanic acid, and those associated with assisted reproductive techniques (e.g., IVF)—were included for their potential effects on vascular function, coagulation pathways, inflammatory modulation, or fetal cardiovascular development. Aspirin and enoxaparin are studied for their roles in modulating placental and fetal blood flow, potentially affecting PDA outcomes. Antibiotics such as cefuroxime and amoxicillin–clavulanic acid were included due to their frequent use in maternal infections, which are known to affect neonatal inflammatory and cardiovascular outcomes.

Missing values were handled using the imputation method, and outliers were reviewed to ensure data consistency.

### 2.2. Statistical Analysis

We initially performed univariate analyses employing logistic regression and chi-square tests to investigate the correlation between maternal factors and the risk of neonatal PDA. This included:

- Logistic regression to identify significant predictors, with computation of odds ratios (OR) and 95% confidence intervals (CIs).
- Chi-square tests to evaluate the significance of associations between categorical maternal variables and PDA occurrence.
- Data preprocessing steps, including categorical variable encoding, handling missing data (imputation method), and outlier analysis.

### 2.3. Machine Learning Models

A Random Forest model was initially created to assess the significance of maternal and medication variables in predicting PDA. The model was trained using 500 trees, employing a subset of features at each split. Feature importance scores were extracted from the RF model to determine the most significant predictors. Subsequent to the Random Forest model, XGBoost was employed to augment predictive precision and assess the impact of each predictor on PDA risk. The parameters of XGBoost, such as learning rate, maximum depth, and subsampling rate, were optimized through cross-validation to mitigate overfitting. The employed objective function was logistic regression for binary classification, utilizing the area under the ROC curve (AUC) as the principal evaluation metric.

Model training and evaluation were performed using standard machine learning validation methods, including stratified splitting of data into training and test subsets, cross-validation, and confusion matrices. Standard methodologies described by Gareth et al. [31], Geron et al. [32] and Kuhn and Johnson [33] were applied to ensure robustness and validity. Confusion matrices were constructed as described by these references, clearly delineating model performance in terms of true positives, false positives, true negatives, and false negatives.

### 2.4. Model Training and Evaluation

The dataset was divided into training (70%) and testing (30%) subsets, applying stratified sampling to preserve the ratio of PDA cases. Cross-validation was conducted within the training set to optimize model parameters and assess model stability. The model’s performance was assessed on the test set utilizing accuracy, sensitivity, specificity, and AUC. Confusion matrices were created to illustrate model performance regarding accurately and inaccurately classified instances. Hyperparameters for both RF and XGBoost were optimized through a grid search employing 5-fold cross-validation to enhance AUC while balancing sensitivity and specificity.

### 2.5. Software and Tools

The study was conducted using R Studio (version 2023.09.1+494 for Mac) with packages *caret*, *xgboost*, and *randomForest* for machine learning modeling. Data preprocessing and visualization were performed using *dplyr* and *ggplot2*. The stats library was utilized for logistic regression (*glm* function) to compute odds ratios and *p*-values, whereas the MASS library was employed for determining confidence intervals for odds ratios. Furthermore, chi-square tests were conducted utilizing the *chisq.test* function within the stats library to assess categorical associations between maternal variables and the presence of PDA. The *dplyr* and *tidyverse* libraries enabled data cleaning and preparation, ensuring precise and efficient data manipulation during the study.

## 3. Results

The study population consisted of 201 neonates admitted to the NICU during the specified period (preterm 69%, and term neonates 31%). Maternal conditions included urinary tract infections, chorioamnionitis, positive endocervical cultures, diabetes, pregnancy-induced hypertension, anemia, prolonged rupture of membranes (>18 h), thyroid disorders, viral infections, and other conditions. The principal pathologies are detailed in Table 1, along with their statistical significance. A total of 89.1% of mothers exhibited at least one pathology during pregnancy, with 95.52% originating from supervised pregnancies (44.28% receiving basic care and 51.24% receiving comprehensive care), while a mere 4.48% were unmonitored pregnancies.

Maternal pathologies, notably prolonged rupture of membranes (PROM), show a strong correlation with increased PDA risk (Odds Ratio: 13.03, *p* < 0.001). This suggests PROM may affect fetal circulatory dynamics, raising PDA risk. Hypertension and diabetes showed moderate, non-significant correlations with PDA; thyroid conditions had no impact. Anemia and IVF showed important odds but had non-significant associations. Medications like enoxaparin and amoxicillin with clavulanic acid slightly increased PDA odds without statistical significance. Methyldopa and cefuroxime did not significantly affect PDA odds. Prenatal care did not influence PDA risk significantly, although high-risk cases might increase its likelihood.

Our analysis confirmed that gestational age and low birthweight significantly predict patent ductus arteriosus (PDA) risk in neonates (Table 2). Each additional week of gestation reduced PDA probability, highlighting preterm infants’ susceptibility due to underdeveloped circulatory systems. Lower birthweight was linked to higher PDA risk. The maternal ages in our cohort ranged from 19 to 42 years, while gestational ages ranged from 28 to 41 weeks. Birth weights varied from 1200 to 4200 g.

### Machine Learning Analysis for PDA Prediction

**Figure 1:**
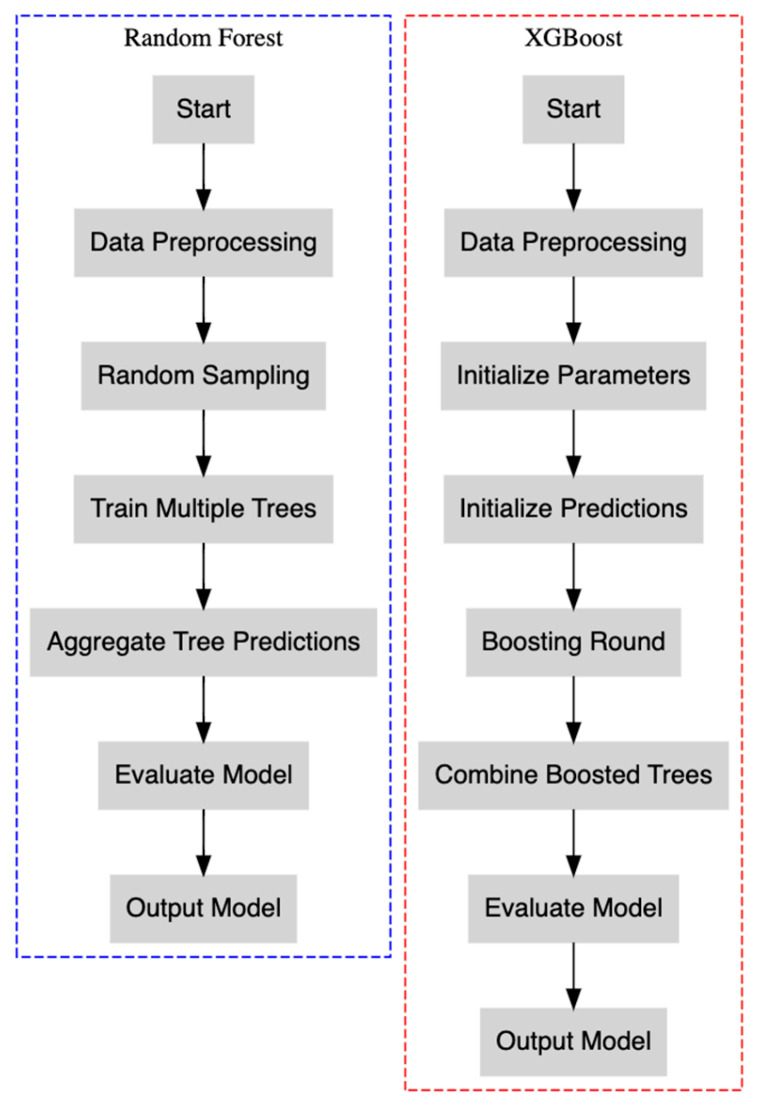
This flowchart encapsulates the procedures involved in both the Random Forest and XGBoost methodologies.

To improve our predictive modeling of PDA risk, we expanded our analysis utilizing machine learning algorithms: Random Forest and XGBoost (Figure 1). These models were selected for their robustness in managing complex, nonlinear interactions and their effectiveness in identifying critical features associated with PDA risk.

**Figure 2:**
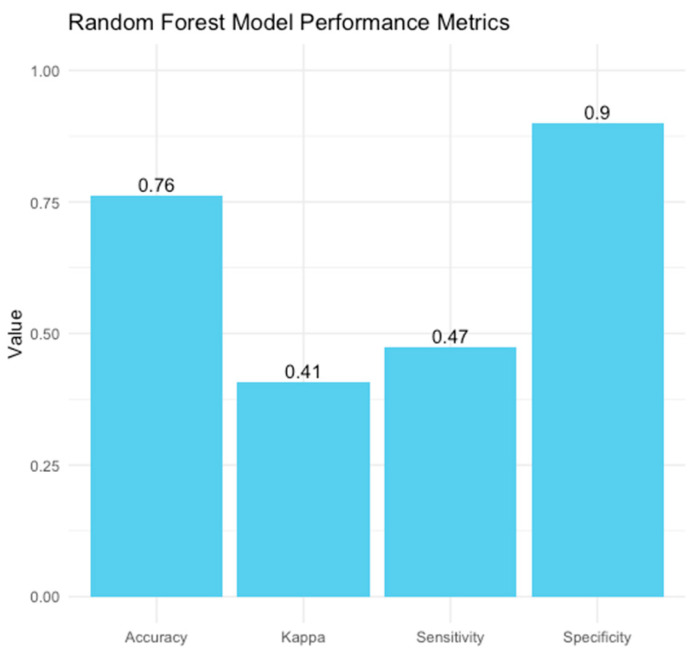
Our RF (Random Forest) performance model.

**Figure 3:**
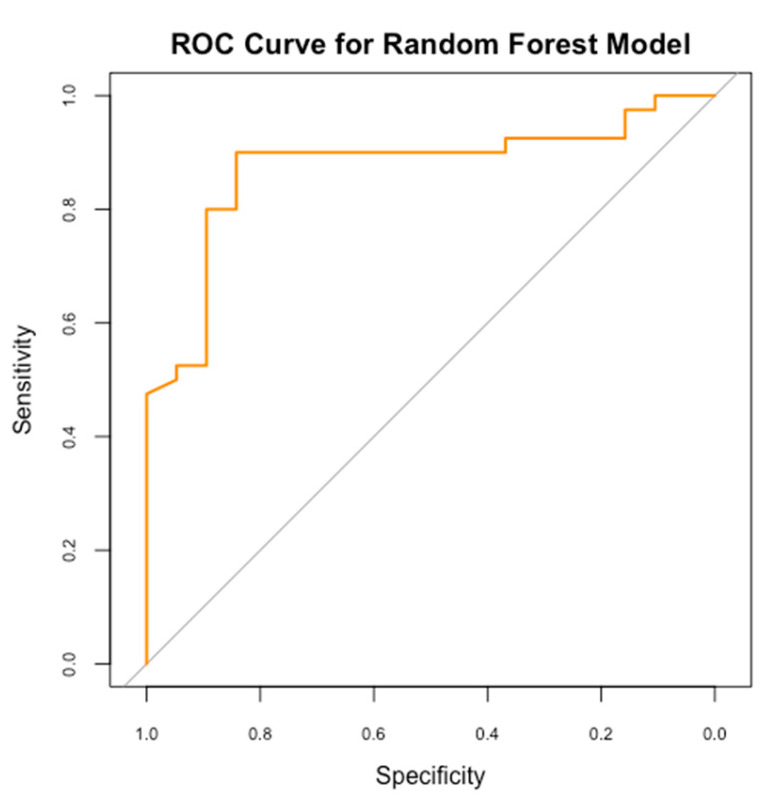
The ROC Curve for our RF performance model.

A Random Forest model was employed to forecast the risk of PDA in neonates, utilizing maternal and neonatal variables. Our model achieved an accuracy of 76.3%, demonstrating moderate efficacy in distinguishing between neonates at high and low risk of PDA. However, the sensitivity was 47.4%, reflecting the model’s ability to detect less than fifty percent of actual PDA cases, indicating limitations in recognizing all at-risk neonates, possibly due to the multifactorial nature of PDA and the variability of risk factors. Moreover, our model’s specificity of 90% reliably identifies neonates without PDA. This indicates that the model effectively minimizes false-positive results, making it valuable for excluding PDA in low-risk scenarios. The Kappa value of 0.41 indicates moderate agreement between predicted and actual classifications, accounting for random chance (Figure 2 and Figure 3).

**Figure 4:**
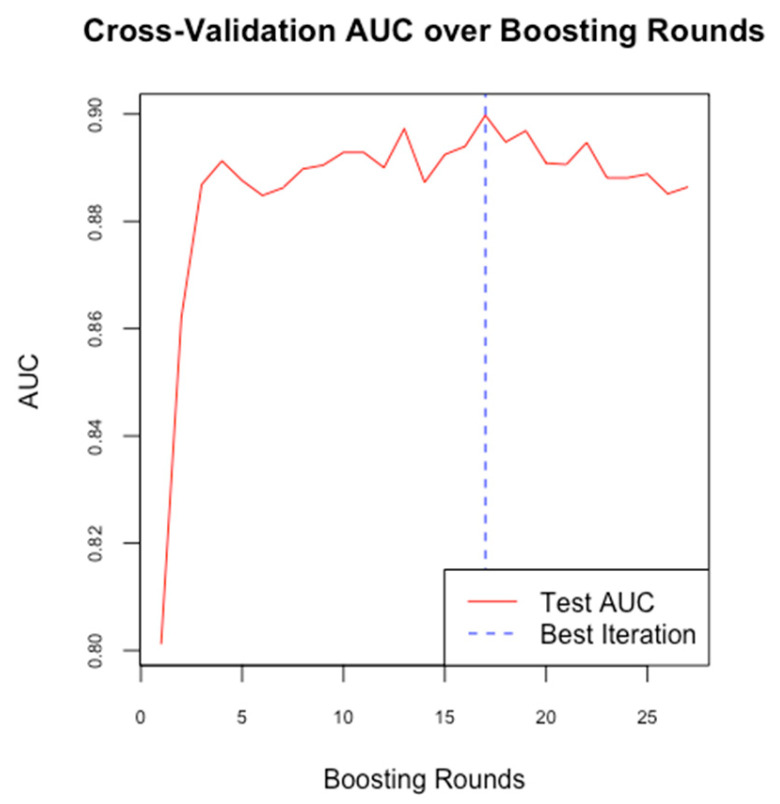
Cross-validation AUC for boosting rounds in our model.

Following the establishment of a baseline model with Random Forest, we implemented the XGBoost algorithm to enhance predictive performance and address the imbalance in sensitivity and specificity observed in the Random Forest model. In our study, we configured XGBoost with optimized hyperparameters, including the learning rate, maximum tree depth, and number of boosting rounds, to improve the model’s accuracy in predicting the likelihood of PDA in neonates. We used cross-validation (Figure 4) to determine the optimal number of boosting rounds and mitigate overfitting, ensuring the model’s effective generalization to new data.

**Figure 5:**
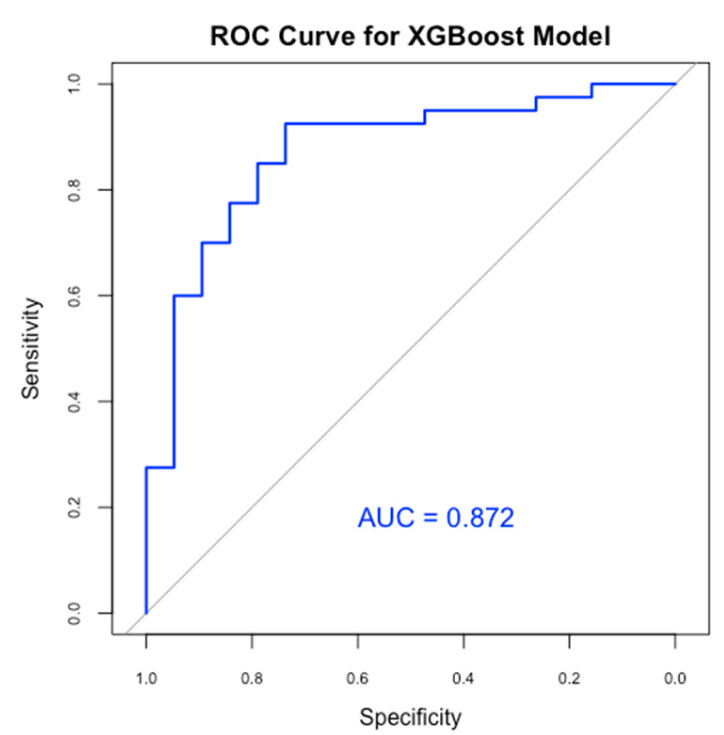
ROC curve for our XGBoost model.

The XGBoost model was used to improve predictive efficacy. Therefore, it achieved an accuracy of 81.4% and an AUC of 0.872 (Figure 5), indicating a strong ability to differentiate between PDA and non-PDA cases. Furthermore, the XGBoost showed a sensitivity of 92.5%, significantly enhancing the detection of true PDA cases, but with a specificity of 57.9%, indicating moderate precision in recognizing non-PDA cases, but a better result compared to the previous model. The Kappa statistic for XGBoost was 0.54, showing improved concordance between predictions and actual results. Our XGBoost model demonstrated superior sensitivity and overall accuracy compared to the Random Forest model, making it more effective for identifying at-risk neonates for PDA.

**Figure 6:**
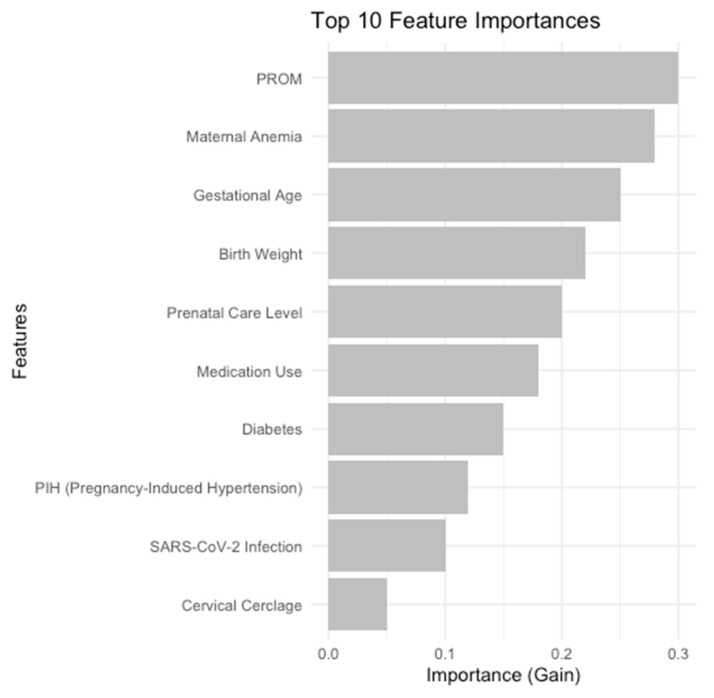
Top 10 predictors provided by our XGBoost model.

Figure 6 provides a bar chart that illustrates the ten most significant features correlated with the probability of Patent Ductus Arteriosus (PDA) in neonates, as determined by the XGBoost model. The primary predictors provide a comprehensive view of the factors affecting PDA risk, emphasizing maternal health conditions, fetal development metrics, and prenatal care as essential components in PDA forecasting.

## 4. Discussion

Our study identified multiple significant maternal and neonatal factors linked to an elevated risk of patent ductus arteriosus in neonates, including prolonged rupture of membranes (>18 h), maternal anemia, gestational age, and birth weight. Lee et al. describe lower gestational age, female gender, and pregnancy-induced hypertension as significant risk factors for symptomatic PDA [34]. PROM surfaced as the paramount predictor, exhibiting a substantial odds ratio and feature significance in both Random Forest and XGBoost models. This association may be attributed to the potential of PROM to precipitate infection or other complications that impact neonatal cardiovascular development. Kusuma et al. concluded that rupture of membranes >12 h and thrombocytopenia in the first 24 h were proven to increase the risk of PDA in preterm infants [35].

Another observation identified gestational age and birth weight as significant predictors, aligning with the existing literature. Low gestational age and low birth weight frequently indicate immature circulatory and pulmonary systems, thereby elevating the risk of PDA [36,37]. Bernati et al. noted that newborns <2000 g at birth tended to have a 1.9 times higher risk for PDA [38].

Maternal anemia demonstrated considerable feature importance, suggesting that inadequate maternal nutrition or oxygenation during gestation may impede fetal development, resulting in neonatal anemia and potentially causing delayed ductal closure, demanding postnatal anemia treatment, as suggested by literature [39,40]. Pregnancy-induced hypertension and diabetes were correlated with elevated odds of patent ductus arteriosus, albeit not consistently with statistical significance. These conditions may indirectly influence fetal cardiovascular development by modifying maternal blood flow and nutrient delivery, thereby affecting the neonate’s capacity to regulate ductal closure [41,42]. Maternal infections, including SARS-CoV-2 and GBS, exhibited a potential association with PDA, although this was not statistically significant. However, infection-induced inflammation may elevate the risk of circulatory complications in neonates, indicating that infection control and maternal prevention could be significant in mitigating PDA risk [43,44].

Extensive prenatal care did not exhibit a significant correlation with the reduction in PDA risk, indicating that standard prenatal care alone may be insufficient to alleviate particular risks associated with PDA, while specialized prenatal interventions aimed at high-risk pregnancies yielded a more significant effect [45]. Medications, specifically enoxaparin and methyldopa, exhibited modest correlations with the risk of PDA. Hoeltzenbein et al. describe a higher rate of major birth defects in methyldopa-exposed pregnancies (3.7%) compared to the cohort (2.5%), but the difference was statistically not significant [46]. Similarly, Bar et al. observed that 3 out of 46 neonates born to mothers undergoing enoxaparin treatment during pregnancy exhibited persistent ductus arteriosus [47].

The ability to predict PDA risk early through machine learning models holds significant clinical importance. Identifying high-risk neonates promptly can facilitate targeted monitoring and immediate therapeutic interventions, potentially reducing complications, hospitalization duration, and related morbidity. Integrating predictive machine learning models systematically into clinical workflows may enhance resource utilization, ensuring optimal neonatal care and individualized treatment planning.

Timely recognition of PDA risk factors, including PROM, reduced gestational age, and maternal anemia, may facilitate targeted interventions, thereby enhancing neonatal outcomes. Healthcare providers may prioritize these factors in prenatal and postnatal care to mitigate the incidence of PDA. For preterm infants with recognized PDA risk factors, enhanced monitoring and management protocols may be instituted to reduce complications related to PDA. These findings may inform clinical decisions regarding antenatal and postnatal care, especially in NICUs that allocate resources to the management of high-risk neonates.

Although our machine learning models demonstrated promising internal validation metrics using stratified training and testing splits, prospective validation on an independent dataset is necessary to confirm the generalizability and clinical utility of the algorithm. Future studies should focus on applying and evaluating these predictive models prospectively in clinical practice to confirm their robustness and improve neonatal outcomes

Our study was constrained by sample size and the omission or incompleteness of certain variables. Subsequent research should target larger cohorts to validate these associations and enhance the predictive accuracy of the models. Further research is required to elucidate the mechanisms connecting maternal health conditions and particular medications to PDA, in order to clarify potential causal relationships. Additional model optimization and evaluation using alternative machine learning techniques, such as neural networks or ensemble methods, could improve predictive accuracy and provide greater understanding of the interactions among maternal, neonatal, and PDA risk factors. The incorporation of machine learning models such as Random Forest and XGBoost in clinical environments may enhance personalized medicine by enabling clinicians to more precisely stratify PDA risk according to specific maternal and neonatal variables.

## 5. Conclusions

This study provides a comprehensive examination of maternal and neonatal factors influencing the risk of patent ductus arteriosus in neonates, combining conventional statistical methods with advanced machine learning techniques. Logistic regression and chi-square tests highlighted prolonged rupture of membranes (>18 h) as a key predictor, while XGBoost identified a broader range of influential variables, including maternal anemia, gestational age, birth weight, and prenatal care level. The results underscore the crucial impact of maternal health and pregnancy-related factors on neonatal outcomes. Future research should confirm these findings in larger, diverse populations and explore using advanced predictive models in clinical decisions to improve neonatal care and outcomes.

## Figures and Tables

**Table 1:** The most important maternal pathologies and medication use from our studied cohort and their significance in PDA risk.

Studied Characteristic	n%	Odds Ratio	95% CI ^2^	*p*-Value
Maternal pathology present	89.1%	1.88	0.77–4.61	0.226
Prolonged rupture of membranes	11.9%	13.03	1.72–98.7	0.00086
Diabetes	11.9%	1.5	0.56–3.98	0.491
GBS ^1^—endocervical culture	1%	0.79	0.44–2.28	0.715
Anemia	5.5%	0.55	0.16–1.89	0.339
IVF ^1^	11.4%	1.83	0.65–5.17	0.344
SARS-CoV-2 infection	3%	2.44	0.28–21.3	0.666
Cervical cerclage	8.5%	1.61	0.50–5.15	0.589
PIH ^1^	16.9%	0.73	0.34–1.57	0.427
UTI ^1^	10.9%	1.71	0.60–4.88	0.347
Autoimmune thyroiditis	2.5%	1.94	0.21–17.7	1
Comprehensive prenatal care	51.24%	1.31	1–1.72	0.429
Amoxicillin + clavulanic acid therapy	20.4%	1.20	0.56–2.53	0.711
Cefuroxime therapy	5.5%	0.83	0.23–2.93	0.749
Methyldopa	14.4%	1.07	0.46–2.50	1
Enoxaparin	9.5%	1.89	0.60–5.94	0.315
Aspirin	3.5%	0.34	0.07–1.58	0.216

^1^ GBS—group B streptococcus, IVF—in vitro fertilization, PIH—pregnancy-induced hypertension, UTI—urinary tract infection, ^2^ CI—confidence interval.

**Table 2:** Maternal age and neonatal risk factors for PDA.

Studied Characteristic	Mean/n%	Odds Ratio	*p*-Value
Maternal age	30.2 years	1.15	0.512
Gestational age	36.5 weeks	0.85	0.042
Birthweight	2.800 g	0.72	0.029
APGAR score	8.1	0.78	0.051
FGR ^1^	14.4%	1.30	0.67

^1^ FGR—fetal growth restriction.

## Data Availability

All data are available in this paper.
